# Early Dental Manifestations and Multidisciplinary Management of X-Linked Hypophosphatemic Rickets in a Pediatric Patient: A Case Report

**DOI:** 10.3390/children13010016

**Published:** 2025-12-20

**Authors:** Nadezhda Mitova, Valentina Petkova-Ninova, Yana Popova

**Affiliations:** 1 Department of Pediatric Dental Medicine, Faculty of Dental Medicine, Medical University, 1431 Sofia, Bulgaria; 2 Department of Pediatric Dentistry, Faculty of Dental Medicine, Medical University of Sofia, 1431 Sofia, Bulgaria; v.petkova@fdm.mu-sofia.bg; 3 Department of Orthodontics, Faculty of Dental Medicine, Medical University of Sofia, 1431 Sofia, Bulgaria; y.popova@fdm.mu-sofia.bg

**Keywords:** X-linked hypophosphatemic rickets, early dental manifestations, spontaneous periapical lesions, pediatric dentistry, multidisciplinary management, orthodontic space maintenance

## Abstract

**Highlights:**

**What are the main findings?**

• Early dental manifestations in children with X-linked hypophosphatemic rickets (XLH), including spontaneous periapical lesions and pulp necrosis in primary teeth, may appear before systemic symptoms and often resist conventional treatment.
• Multidisciplinary management, integrating pediatric dentistry, orthodontics, and endocrinology, enables timely systemic therapy and dental preservation.

**What are the implications of the main findings?**

• Careful dental assessment of atypical, caries-free lesions can facilitate the early diagnosis of phosphate metabolism disorders.
• Interdisciplinary collaboration helps optimize dental and systemic outcomes, including reduced extractions and maintenance of arch integrity.

**Abstract:**

**Background:** X-linked hypophosphatemic rickets (XLH) is a rare hereditary disorder characterized by renal phosphate wasting and impaired bone mineralization. Oral manifestations such as spontaneous periapical lesions and dental abscesses in the absence of caries or trauma may precede systemic features in XLH due to underlying dentin hypomineralization and enamel–dentin junction defects, and could serve as early diagnostic indicators. **Case Report:** We report on the case of a 4-year-old boy referred to our pediatric dental unit with recurrent intraoral fistulas persisting over the past year. Clinical examinations and an orthopantomogram revealed extensive root resorption and periapical pathology affecting multiple primary molars without evident caries or trauma. Laboratory investigations showed hypophosphatemia, elevated renal phosphate loss, and raised inflammatory markers (CRP (C-reactive protein) and granulocytes). Genetic testing of the child and his mother confirmed a diagnosis of X-linked hypophosphatemic rickets. **Management:** Due to behavioral challenges, treatment proceeded with difficulty over multiple visits. Endodontic treatment was initiated using a formalin–resorcinol technique; however, several primary molars developed progressive necrosis and required extraction. Orthodontic space maintainers were placed to preserve arch integrity and support future eruption. The patient remains under follow-up and is currently awaiting Burosumab therapy. Despite systemic management, spontaneous necroses of the primary molars persist, highlighting the refractory nature of dental involvement in XLH. **Conclusions:** This case underscores the pivotal role of pediatric dentists in recognizing systemic diseases through oral findings and demonstrates the challenges of managing XLH-related dental pathology, even under targeted systemic therapy. Early interdisciplinary collaboration is essential to optimize both dental and systemic outcomes in affected children.

## 1. Introduction

Hypophosphatemic rickets (HR) is a metabolic disorder affecting the bones and teeth. X-linked hypophosphatemic rickets is the most common form and is characterized by skeletal deformities and dental mineralization defects, leading to pulpitis and early tooth loss. The initial complaints are a delay in the development of walking, caused by a deformity of the legs. Oral findings include poorly mineralized dentin, enlarged pulp chambers and root canals, and periradicular abscesses in caries-free teeth [1]. Early diagnosis and preventive interventions are crucial for maintaining oral health in affected children [2,3,4].

Early diagnosis, optimal management, and regular follow-up of children with XLH determine long-term outcomes and quality of life. Monitoring includes clinical, biochemical, and radiological assessments, alongside screening for XLH-related complications. In 2018, the EU approved Burosumab, a humanized monoclonal anti-FGF23 (fibroblast growth factor 23) antibody, as an alternative therapy for growing children with insufficiently controlled XLH [5].

Loss-of-function mutations in the PHEX gene (phosphate-regulating gene with homologies to endopeptidases on the X chromosome) elevate circulating FGF23, causing renal phosphate waste, hypophosphatemia, and impaired vitamin D metabolism. These biochemical abnormalities disrupt phosphate and bone mineralization, leading to rickets in children; osteomalacia in adults; poor growth, including persistent short stature; skeletal deformities; pain; stiffness; dental complications; and compromised physical function [6,7].

However, oral manifestations, such as spontaneous dental abscesses, pulp necrosis, enlarged pulp chambers, and root resorption, may precede systemic symptoms. These atypical dental signs frequently occur in the absence of dental caries or trauma, posing a diagnostic challenge. Although the majority of XLH diagnoses are made after the appearance of systemic signs, there is growing recognition in the literature that pediatric dentists can be the first healthcare professionals to identify the disorder based on oral findings [2,3]. The management of XLH requires a multidisciplinary team, ideally coordinated by a metabolic bone disease specialist, to ensure comprehensive care across pediatric and adult stages [8].

In 2018, the EU approved Burosumab, a humanized monoclonal anti-FGF23 (fibroblast growth factor 23) antibody, as an alternative therapy for growing children with insufficiently controlled XLH; it has been shown to normalize phosphate metabolism and prevent further height deficit in affected toddlers [5,9].

Approximately 10–20% of XLH cases may be initially diagnosed through dental presentations, highlighting the importance of dental vigilance and interdisciplinary collaboration. This case report demonstrates how early dental findings can prompt diagnosis and guide targeted multidisciplinary care.

This case is notable because the diagnosis of XLH was prompted exclusively by atypical, caries-free dental findings (recurrent fistulas and spontaneous periapical lesions) before the occurrence of any musculoskeletal symptoms, underlining the role of pediatric dentists in presymptomatic detection—a diagnostic pathway less frequently documented in the literature.

## 2. Case Presentation

**Patient Information:** The patient was a 4-year-old boy born from a third uneventful pregnancy via planned term cesarean section (undertaken due to a nuchal cord and maternal indications), with a birth weight of 3380 g and length of 52 cm. The neonatal period was uncomplicated. After 2 months of age, he was fed with an adapted formula. He started walking at 1 year of age. From birth until 2 years, he received vitamin D supplementation (1 drop of 400 IU/day). His diet is varied, including meat and dairy products.

**Family History:** Mother: height 162 cm, menarche at 13–14 years, exhibits pectus carinatum and genu valgum (H-bow) deformity, which was more pronounced in childhood. Father: height 178 cm, reports no relatives with bone disorders, short stature, or limb deformities.

**Past Medical History**: Surgery for funiculocele at 1 year and 2 months and at 2 years; a single episode of bronchiolitis at 1 year and 8 months.

**Figure 1:**
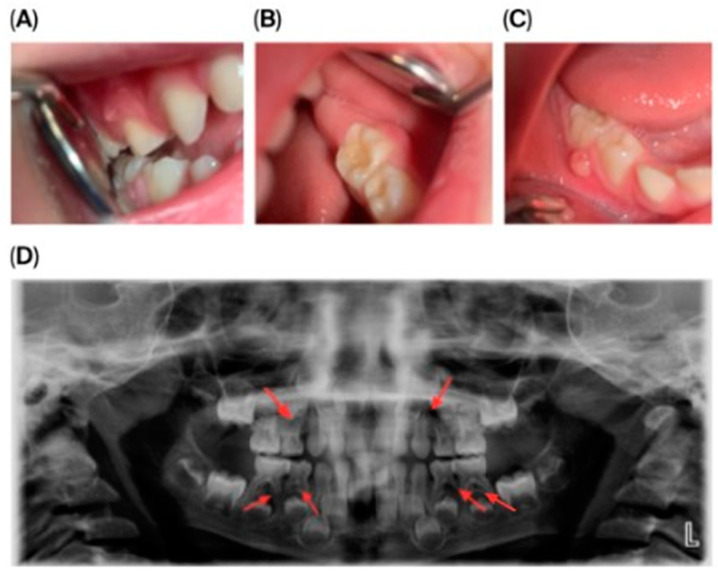
**Intraoral and extraoral photographs and panoramic imaging of the patient showing multiple fistulas and dental pathology**: (**A**) Buccal fistula in the upper right quadrant. (**B**) Buccal fistula in the lower left quadrant. (**C**) Buccal fistula in the lower right quadrant. (**D**) Panoramic radiograph (2024) showing extensive periapical radiolucency and root resorption in multiple primary molars (54, 64, 74, 75, 84, 85).

**Dental Findings: Clinical and radiographic aspects:** Intraoral examination revealed four fistulous openings on the buccal mucosa, located adjacent to the upper left first primary molar (tooth 64), upper right first primary molar (tooth 54), lower left second primary molar (tooth 75), and lower right second primary molar (tooth 85). The gingiva appeared normal, and there were no carious lesions or signs of trauma (Figure 1A–D).

Panoramic radiography (Figure 1D) showed pronounced internal and external root resorption and extensive periapical radiolucency involving several primary molars (54, 64, 74, 75, 84, 85). The lesions measured approximately 2–4 mm, were diffuse, mainly affecting the furcation area and the medial roots, and displayed moderate, irregularly shaped root resorption. More advanced changes were observed in teeth 64, 74, and 85. The permanent tooth germs were intact, and no abnormalities of the eruption pattern or mineralization were noted at this stage.

**General Findings:** Somatic evaluation revealed no characteristic manifestations of rickets, no skeletal abnormalities, and normal gait and body proportions. Radiographs of the wrist indicated a bone age of approximately 5 years and 8 months. The joint spaces of both knees were normally wide. Bone structures forming the joints appeared normal, with smooth and sharp contours. No radiographic evidence of rickets. A pediatric nephrology consultation and abdominal ultrasound were performed.

**Laboratory and Genetic Investigations:** Initial laboratory tests revealed hypophosphatemia (0.68 mmol/L) with increased urinary phosphate excretion. Serum calcium was within normal range, alkaline phosphatase was mildly elevated, and parathyroid hormone was within upper-normal limits. Vitamin D levels were adequate, while inflammatory markers were elevated, reflecting chronic periapical inflammation. Based on these findings and the clinical presentation, molecular genetic testing of the PHEX gene was performed, identifying a hemizygous c.1483-1G>T mutation, confirming a diagnosis of XLH. Maternal testing demonstrated heterozygous carrier status, establishing a familial predisposition.

**Treatment and Dental Management:** Dental treatment was initiated in May 2024, approximately one month before the formal diagnosis of XLH was established. The pediatric dental team was the first to suspect an underlying metabolic disorder based on atypical, caries-free periapical lesions, which prompted early referral to pediatric endocrinology and clinical genetics. Burosumab therapy was approved and initiated in August 2024 after completion of the diagnostic evaluation and multidisciplinary review.

Conventional therapy was initiated with phosphate supplementation (Phosphate Sandoz 500 mg, 4 × 1/3 tablet dissolved in water) and One Alfa 2 mg/mL solution (4 drops daily) until approval for Burosumab therapy. Burosumab (0.8 mg/kg subcutaneously every two weeks) was started on 20 August 2024, when the patient was 5 years old, alongside daily vitamin D supplementation (800 IU), with parental training for subcutaneous administration. An improvement in serum phosphate levels and tubular reabsorption of phosphate (TmP/GFR) was observed over the first few weeks, indicating a positive systemic response. The results are summarized in Table 1.

**Figure 2:**
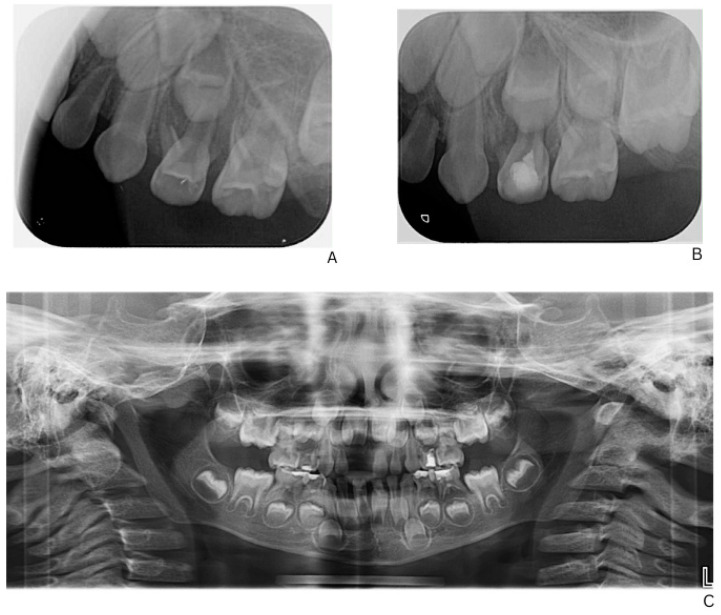
**Radiographic follow-up of upper left first primary molar showing initial and post-treatment periapical status, and panoramic overview**. (**A**) Pre-treatment periapical radiograph of the upper left first primary molar. (**B**) Post-treatment periapical radiograph showing healing after formalin–resorcinol endodontic protocol and glass–ionomer cement restoration. (**C**) Panoramic radiograph (OPG, 2025) demonstrating overall progression of dental treatment and status of other primary molars.

Initial dental management focused on infection control and the preservation of dental function. A formalin–resorcinol endodontic protocol was applied to the affected primary molars, followed by long-term glass–ionomer cement restorations (VOCO GmbH, Cuxhaven, Germany) (Figure 2). Due to the child’s uncooperative behavior, treatment required multiple short appointments with behavioral guidance. The clinical team considered that the behavior could be gradually managed, and in agreement with the parents’ informed preference, treatment was carried out in short, sequential sessions rather than under general anesthesia or sedation.

Given the patient’s limited cooperation, the lack of alternative methods capable of effectively controlling the periapical inflammation in this specific pathogenic context, and the need to maintain the primary teeth until their physiological exfoliation, a resorcinol–formalin technique was selected. This approach represented the only practically effective option in a situation characterized by spontaneously developing pulp necroses in clinically intact teeth.

**Follow-up and Outcome:** At the most recent follow-up (12 months after initial presentation), most endodontically treated primary molars remained stable, with occasional mild gingival inflammation resolving with oral hygiene.

The lower right second primary molar (tooth 85) was initially observed following stabilization of the acute condition and completion of endodontic treatment of the other primary molars. At reassessment, radiographic evaluation revealed advanced and rapidly progressing root resorption, particularly affecting the mesial root, rendering endodontic therapy contraindicated and without reasonable prognosis. Consequently, extraction was performed to prevent further complications.

The patient sustained dental trauma resulting in the early loss of 51 (upper right primary central incisor). The tooth was avulsed, and the parents were informed about the incident. Home-care instructions were provided, and follow-up appointments were scheduled. Due to the young age of the patient and limited cooperation, an anterior space maintainer was not placed at that time, although future orthodontic evaluation will consider space maintenance if indicated.

**Figure 3:**
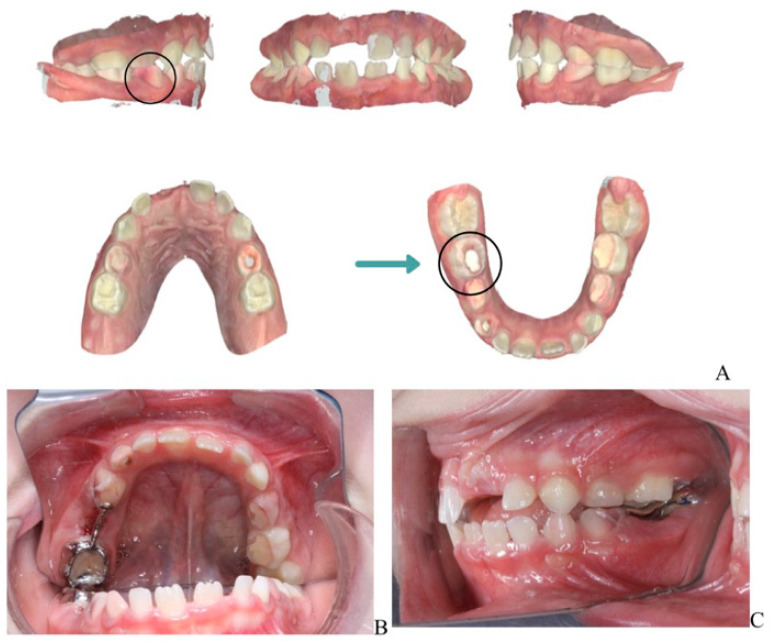
**Orthodontic management following extraction of mandibular right second primary molar (tooth 85).** (**A**) Scanned models of the dental arch showing the area of extraction and planned space maintenance. (**B**) Indirect intraoral mirror photograph showing the laser-sintered, digitally designed space maintainer cemented in situ to preserve arch integrity and guide eruption of the permanent successor (tooth 45). (**C**) Indirect intraoral mirror photograph showing a lateral view of the space maintainer, illustrating its position and adaptation to the adjacent teeth.

A laser-sintered digitally designed space maintainer was fabricated and cemented to preserve arch integrity and guide the eruption of the permanent successor (tooth 45) (Figure 3). Ongoing monitoring includes dental, biochemical, and renal assessments every 3–6 months.

**Figure 4:**
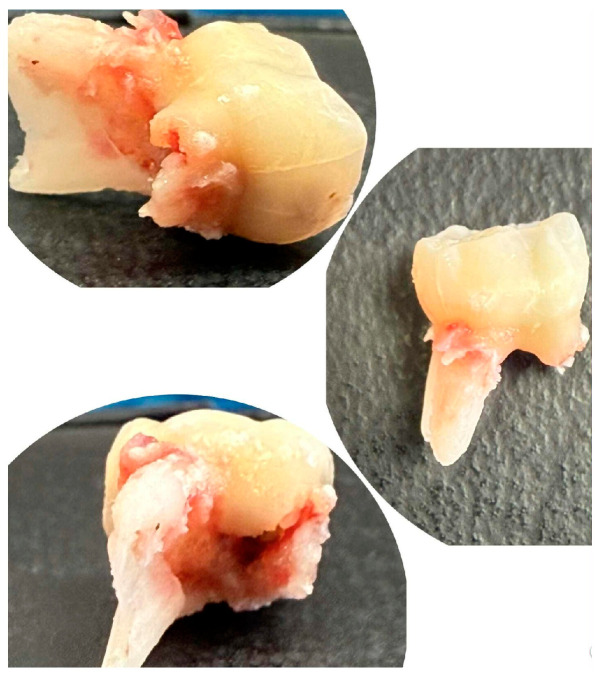
Extracted mandibular right second primary molar (tooth 85).

The extracted lower right second primary molar (tooth 85) is shown in Figure 4, demonstrating the extent of periapical pathology, with more than two-thirds resorption of the mesial root and structural changes.

**Figure 5:**
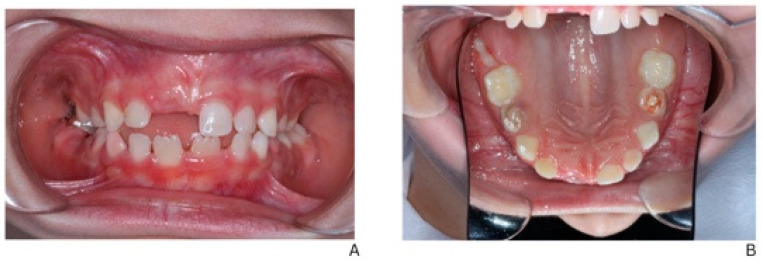
Post-treatment intraoral view showing regression of fistulas and stable gingival health. (**A**) Intraoral frontal view in occlusion. (**B**) Occlusal view of the maxilla.

Figure 5 illustrates the post-treatment intraoral condition of the patient. Regression of the previously noted fistulas is evident, and the gingival tissues appear healthy with no signs of inflammation. This demonstrates the effectiveness of the staged dental interventions and ongoing management.

**Systemic and Interdisciplinary Management:** Following XLH confirmation, the patient was enrolled in a multidisciplinary care program, including pediatric endocrinology, dentistry, orthodontics, and clinical genetics. Burosumab therapy was initiated to correct phosphate metabolism, improve skeletal mineralization, and potentially reduce dental complications. The treatment plan prioritizes early dental intervention to prevent abscess recurrence, maintain oral function, and coordinate with systemic therapy.

These systemic and dental observations will be further discussed in the context of the existing literature in the Section 3.

**Summary:** This case underscores the central role of pediatric dentists in the early recognition of XLH through atypical dental manifestations, enabling timely genetic diagnosis and the initiation of targeted therapy. It also highlights the importance of early and precise pediatric dental management, as persistent dental pathology may not respond to conservative treatment, making timely intervention crucial for preserving function and guiding the eruption of permanent dentition. Multidisciplinary coordination with endocrinology, orthodontics, and genetics ensures optimal oral and systemic outcomes.

## 3. Discussion

X-linked hypophosphatemic rickets is the most common hereditary form of rickets, caused by mutations in the PHEX gene and characterized by renal phosphate wasting and impaired bone mineralization [5]. Typical clinical features include bone deformities, short stature, delayed growth and dental abnormalities, although systemic manifestations may be absent in early childhood [10,11,12].

In our patient, dental involvement, spontaneous periapical abscesses, pulp necrosis in clinically intact teeth, and early root resorption align closely with the characteristic dental phenotype of XLH [1,2,3]. These manifestations reflect the underlying dentin hypomineralization [13] and enamel–dentin junction defects described in the literature [13], and they represent the earliest signs of the disorder in this child [2].

In the present case, the first and only presenting signs were recurrent intraoral fistulas and unexplained periapical lesions in the absence of caries or trauma [1,3]. These atypical dental findings prompted the pediatric dentist to initiate further investigations, including biochemical and genetic testing, which revealed hypophosphatemia and increased renal phosphate loss and ultimately confirmed the diagnosis of XLH before musculoskeletal symptoms appeared [12]. Genetic analysis also identified the condition in the mother, confirming hereditary transmission [7]. XLH poses significant challenges for dental management, as many patients develop spontaneous abscesses and sinus tracts affecting both primary and permanent dentition [11].

The dental management of XLH is challenging and often controversial [12]. Some authors recommend extraction of teeth with recurrent periradicular abscesses and implant restoration, as endodontic and restorative treatments often fail to maintain long-term asepsis [13]. Localized antibiotic therapy and chlorhexidine rinses may help to control soft tissue inflammation, although dental abscesses remain one of the most reported complications [1,14].

Some authors discuss extraction and implant placement in adults with XLH; however, in pediatric patients, the focus is on preserving primary teeth and supporting permanent dentition [1,12,13]. Extraction is generally considered only for severely compromised teeth with recurrent abscesses or advanced structural defects [11,13,15]. In this case, tooth 85 was extracted due to persistent periapical pathology and extensive root resorption, consistent with the limited healing potential of affected primary teeth reported in the literature [13,15].

The choice of resorcinol–formalin in this case was guided by clinical constraints and the pathogenesis of pulp necrosis in XLH [15]. Unlike materials such as Biodentine or MTA, which primarily support hard tissue formation and have limited direct antimicrobial activity, the resorcinol–formalin protocol chemically fixes remaining pulp tissue, forming a bakelite-like mass with antiseptic properties [14]. LSTR (Lesion Sterilization and Tissue Repair) represents the closest mechanistic alternative; however, its effectiveness is considerably lower in cases of spontaneously occurring necroses in clinically intact teeth, as seen in XLH [15]. In the context of poor behavioral cooperation and the need to preserve the primary dentition until physiological exfoliation, the resorcinol–formalin technique was the most reliable option [14,15].

Data on non-vital endodontic therapies in primary teeth remain mixed, with some studies reporting favorable clinical outcomes [16] while others demonstrate limited radiographic healing or recurrent pathology depending on the filling material and technique used [17]. This variability underscores the need for case-specific decision making, especially in conditions such as XLH, where the pathogenesis differs from typical caries-related pulp disease [16,17].

Treatment response varied among affected teeth, reflecting the initial severity of structural defects [13] and bacterial invasion [13]. The only tooth that failed to stabilize was also among those exhibiting the most advanced periapical radiolucency and extensive root resorption at baseline, which is consistent with previous observations that severely compromised primary teeth in XLH have limited healing potential regardless of the endodontic technique used [13].

Given that the permanent tooth germs were radiographically intact and that systemic therapy with Burosumab was initiated before the onset of skeletal symptoms, the prognosis for the permanent dentition in this child is cautiously favorable. Early correction of phosphate metabolism may support improved dentin mineralization compared with the primary teeth. However, the risk of pulp complications cannot be entirely excluded, and long-term surveillance will be required to monitor the development of permanent dentition [18].

XLH is an X-linked dominant disorder, with more severe skeletal and dental manifestations reported particularly in male patients. Dental involvement may affect both primary and permanent dentitions. Conventional treatment consists of the administration of active vitamin D analogues and oral phosphate supplements, aiming to correct phosphate homeostasis and support overall mineralization, including dentin formation [12,13,19]. Loss of PHEX function in odontoblasts directly leads to dentin hypomineralization, predisposing affected teeth to spontaneous pulp necrosis and periapical pathology [12,13]. Early initiation of systemic therapy and close pediatric monitoring are therefore considered important to optimize dental outcomes. In many patients, spontaneous periapical abscess formation in early childhood represents the first clinical sign leading to the diagnosis of XLH [12,13].

Conventional management of X-linked hypophosphatemia relies on long-term combined therapy, which is typically administered three to five times daily and may pose challenges to treatment adherence, particularly in school-aged children. These interventions aim to support dentin recovery and overall mineralization, as demonstrated in experimental models and clinical studies [6,19]. Despite treatment, responses are often incomplete, with persistent metaphyseal changes, progressive lower limb deformities, and poor growth resulting in short stature. In adults, XLH may cause persistent bone and joint pain, enthesitis, insufficiency fractures, spinal entrapment, and recurrent dental abscesses, often leading to extractions; deafness prevalence also increases with age [13,20].

Vitamin D is a steroid hormone that increases serum calcium and phosphate and promotes mineralization of the bone matrix and growth plate through its active metabolite, calcitriol (1,25(OH)_2_D). In the skin, 7-dehydrocholesterol is converted to cholecalciferol (vitamin D_3_) through a series of enzymatic reactions. Phosphate absorption in the small intestine occurs predominantly via passive mechanisms (85%) and partly through the sodium-dependent phosphate transporter NaPi-2b (SLC34A2). In the kidneys, particularly in the proximal tubules, phosphate reabsorption is mediated by NaPi-2a (SLC34A1) and NaPi-2c (SLC34A3), regulated by PTH and FGF23. GF23 inhibits these transporters, increases urinary phosphate excretion, and suppresses active vitamin D synthesis, thereby reducing intestinal calcium and phosphate absorption [19].

The mechanism by which PHEX loss of function leads to elevated levels of circulating intact FGF23 remains unclear. Rickets results from reduced serum calcium and/or phosphate levels, leading to impaired and delayed mineralization of the growth plates. In some hereditary forms, renal phosphate wasting is due to mutations in phosphate transporters, such as in hypophosphatemic rickets with hypercalciuria or Fanconi syndrome. PHEX, the gene responsible for XLH, encodes a cell surface-bound endopeptidase expressed mainly in the bone and teeth. Tooth eruption may be delayed and enamel often appears clinically normal. Impaired dentin mineralization is characteristic and predisposes patients to spontaneous dental abscesses and early decay in both primary and permanent dentitions [13,19].

This case underscores the critical importance of oral findings in the early detection of systemic diseases [1]. Dental manifestations such as spontaneous abscess formation, pulp necrosis, and root resorption in the absence of caries are well-documented features of XLH, yet they are frequently overlooked [1]. In this patient, the pediatric dentist’s recognition of these atypical signs prompted referral to endocrinology, enabling the timely initiation of Burosumab therapy [21]. Early diagnosis is essential in XLH, as delayed treatment can lead to significant skeletal deformities, functional impairment, and reduced quality of life [20]. This case highlights how early pediatric dental assessment can serve as a frontline tool for detecting systemic disease and facilitating pre-symptomatic medical intervention [21].

The diagnostic pathway in this case highlights the importance of interdisciplinary collaboration between dentistry, endocrinology, and genetics, particularly for children presenting with atypical dental pathology [22]. In 2018, Burosumab, a fully human monoclonal antibody against FGF23, was approved for XLH treatment and has been shown to normalize serum phosphorus, reduce rickets severity, and improve growth and physical function in children [23,24]. Endodontic treatment may represent a first-line therapeutic option in selected cases, particularly when teeth are structurally preserved and periapical pathology is manageable, although outcomes remain variable in XLH due to underlying dentin hypomineralization [11,13,25]. However, dental manifestations, especially pulp necrosis and periapical pathology in primary teeth, may respond less predictably to systemic therapy, as illustrated by our patient [25,26]. This emphasizes the need for targeted dental management, including timely extractions, preventive measures, and space maintenance, to support oral function and long-term outcomes [25,26].

Comprehensive and early dental intervention is essential to minimize functional and developmental complications in pediatric XLH patients. Appropriate dental care, combined with systemic treatment, can support radiological healing of rickets and improve lower-limb alignment, although growth outcomes may vary. Future research should assess early Burosumab therapy on dentin and permanent tooth development, refine dental management to limit extractions and preserve function, and examine long-term outcomes of interdisciplinary care [27].

**Limitations:** This case has several limitations. First, as a single-patient report, the findings cannot be generalized to all children with XLH. Second, although early dental treatment began before the genetic confirmation, the lack of skeletal manifestations at presentation limits the assessment of correlations between dental findings and systemic disease severity. Third, the follow-up period remains relatively short, which restricts conclusions regarding long-term outcomes, particularly for the permanent dentition. Finally, histopathological examination of the extracted primary tooth was not available, which would have provided additional information on dentin structure.

## 4. Conclusions

This case underscores the critical role of pediatric dentists in identifying atypical dental signs that may be suggestive of underlying metabolic or genetic conditions such as XLH, enabling timely systemic therapy and preventive planning. Early interdisciplinary collaboration with endocrinology and genetics is essential to optimize both dental and systemic outcomes. Continuous monitoring and targeted dental management help preserve oral function, guide permanent dentition, and minimize long-term complications.

## Figures and Tables

**Table 1:** Serum phosphate (P) and tubular maximum reabsorption of phosphate per glomerular filtration rate (TmP/GFR) over the follow-up period.

	3 September 2024	20 September 2024	5 October 2024
P, mmol/L (1.05–1.8)	1.34	1.18	1.19
TmP/GFR (1.05–1.78)	1.20	1.01	1.09

## Data Availability

The data presented in this study are available on request from the corresponding author. The data are not publicly available due to privacy restrictions.

## Abbreviations

The following abbreviations are used in this manuscript:

XLH	X-linked hypophosphatemic rickets
HR	Hypophosphatemic rickets
CRP	C-reactive protein
FGF23	Fibroblast growth factor 23
PHEX	Phosphate-regulating gene with homologies to endopeptidases on the X chromosome
TmP/GFR	Tubular maximum reabsorption of phosphate per glomerular filtration rate
NaPi-2a	Sodium-dependent phosphate transporter 2a
NaPi-2b	Sodium-dependent phosphate transporter 2b
NaPi-2c	Sodium-dependent phosphate transporter 2c
PTH	Parathyroid hormone
VDR	Vitamin D receptor
